# Activation of AMPK/SIRT1/FOXO3a signaling by BMS-477118 (saxagliptin) mitigates chronic colitis in rats: uncovering new anti-inflammatory and antifibrotic roles

**DOI:** 10.3389/fphar.2024.1456058

**Published:** 2024-09-18

**Authors:** Elsayed A. Elmorsy, Mahmoud E. Youssef, Mohamed R. Abdel-Hamed, Maha M. Amer, Sahar R. Elghandour, Abdullah S. Alkhamiss, Nahla B. Mohamed, Mostafa M. Khodeir, Hossam A. Elsisi, Thamir Saad Alsaeed, Manal M. Kamal, Abousree T. Ellethy, Basem H. Elesawy, Sameh Saber

**Affiliations:** ^1^ Department of Pharmacology and Therapeutics, College of Medicine, Qassim University, Buraidah, Saudi Arabia; ^2^ Department of Pharmacology, Faculty of Pharmacy, Delta University for Science and Technology, Gamasa, Egypt; ^3^ Department of Anatomy, College of Medicine, Qassim University, Buraidah, Saudi Arabia; ^4^ Department of Anatomy and Embryology, Faculty of Medicine, Ain Shams University, Cairo, Egypt; ^5^ Department of Anatomy and Histology, College of Medicine, Qassim University, Buraidah, Saudi Arabia; ^6^ Department of Pathology, College of Medicine, Qassim University, Buraidah, Saudi Arabia; ^7^ Department of Pathology, Faculty of Medicine, Cairo University, Cairo, Egypt; ^8^ Department of Pharmacology and Toxicology, College of Pharmacy, Qassim University, Buraidah, Saudi Arabia; ^9^ Department of Clinical Pharmacology, Faculty of Medicine, Zagazig University, Zagazig, Egypt; ^10^ Department of Biology and Immunology, College of Medicine, Qassim University, Buraidah, Saudi Arabia; ^11^ Department of Medical Physiology, Faculty of Medicine, Assiut University, Assiut, Egypt; ^12^ Department of Physiology, College of Medicine, Qassim University, Buraidah, Saudi Arabia; ^13^ Department of Oral and Medical Basic Sciences, Biochemistry Division, College of Dentistry, Qassim University, Buraidah, Saudi Arabia; ^14^ Department of Pathology, College of Medicine, Taif University, Taif, Saudi Arabia; ^15^ Department of Pathology, Faculty of Medicine, Mansoura University, Mansoura, Egypt

**Keywords:** BMS-477118 (saxagliptin), AMPK/SIRT1/FOXO3a, chronic ulcerative colitis, intestinal inflammation/fibrosis, DPP4 inhibitors, novel therapeutic target

## Abstract

Ulcerative colitis (UC) is a debilitating chronic disease marked by persistent inflammation and intestinal fibrosis. Despite the availability of various treatments, many patients fail to achieve long-term remission, underscoring a significant unmet therapeutic need. BMS-477118, a reversible inhibitor of dipeptidyl peptidase 4 (DPP4), has demonstrated anti-inflammatory properties in preclinical and clinical studies with minimal adverse effects compared to other antidiabetic agents. However, the potential benefits of BMS-477118 in chronic UC have not yet been explored. In this study, we aimed to investigate the effects of BMS-477118 in rats subjected to chronic dextran sodium sulfate (DSS) administration. Our findings indicate that BMS-477118 activates the interconnected positive feedback loop involving AMPK, SIRT1, and FOXO3a, improving histological appearance in injured rat colons. BMS-477118 also reduced fibrotic changes associated with the chronic nature of the animal model, alleviated macroscopic damage and disease severity, and improved the colon weight-to-length ratio. Additionally, BMS-477118 prevented DSS-induced weight loss and enhanced tight junction proteins. These effects, in conjunction with reduced oxidative stress and its potential anti-inflammatory, antiapoptotic, and autophagy-inducing properties, fostered prolonged survival in rats with chronic UC. To conclude, BMS-477118 has the potential to activate the AMPK/SIRT1/FOXO3a signaling pathway in inflamed colons. These results suggest that the AMPK/SIRT1/FOXO3a pathway could be a new therapeutic target for UC. Further research is mandatory to explore the therapeutic possibilities of this pathway. Additionally, continued studies on the therapeutic potential of BMS-477118 and other DPP4 inhibitors are promising for creating new treatments for various conditions, including UC in diabetic patients.

## 1 Introduction

Ulcerative colitis (UC) is a chronic inflammatory bowel disease (IBD) of unspecified etiology (Tan et al., 2024). Genetic background, aberrant immune responses, and exposure to environmental and luminal factors have been suggested to contribute to UC pathogenesis (Abdelhady et al., 2023). In 2023, the global prevalence of UC was estimated at 5 million cases. Additionally, the incidence continues to rise worldwide (Le Berre et al., 2023). This highlights the significance of research in prevention and healthcare innovations (Wang et al., 2024). Unfortunately, current medical treatment for chronic UC aims primarily at preventing disease complications (Abdelhamid et al., 2022). Moreover, the clinical outcomes of existing therapies are often inadequate (Zohny et al., 2022a). Furthermore, patients who do not respond to these treatments frequently require hospitalization. Therefore, it is crucial to develop new medications with innovative mechanisms of action to improve patient outcomes (Saber et al., 2021; Cavalu et al., 2022; Abdelhady et al., 2023).

The natural progression of UC involves a complex interplay of several cellular processes that collectively contribute to the disease’s pathophysiology (Nasr et al., 2022). During the course of UC, genetic predispositions play a significant role in disrupting the integrity of the gastrointestinal epithelium. This disruption compromises the mucosal barrier, making the colon more susceptible to inflammatory assaults (Saber et al., 2021). As a result, the immune system’s response becomes dysregulated, leading to chronic inflammation within the colonic mucosa (Kaur and Goggolidou, 2020). Several key cellular mechanisms become dysregulated in UC, exacerbating the disease. For instance, sirtuin-1 (SIRT1) normally helps to regulate inflammation and oxidative stress in the gut. However, in UC, the protective functions of SIRT1 are often overwhelmed, leading to unchecked inflammatory responses and tissue damage (Caruso et al., 2014; Yang et al., 2022b). Another critical player is adenosine monophosphate-activated protein kinase (AMPK), an energy sensor that, under normal conditions, helps maintain cellular energy balance and promotes autophagy. In UC, however, AMPK activity may be insufficient, contributing to the accumulation of cellular debris and further aggravating inflammation (Cheng et al., 2024). Autophagy itself is a crucial process for maintaining intestinal homeostasis by clearing damaged organelles and pathogens. In UC, impaired autophagy disrupts the function of intestinal epithelial cells, leading to a compromised epithelial barrier, alterations in the gut microbiota, and heightened inflammation (Foerster et al., 2022). Apoptosis also plays a significant role in the natural course of UC. Excessive apoptosis of intestinal epithelial cells can lead to the thinning of the intestinal lining, worsening barrier dysfunction, and facilitating the persistence of inflammation (Martínez et al., 2012). Conversely, inadequate apoptosis may allow damaged cells to survive, further contributing to the inflammatory milieu and disease progression.

Inhibition of dipeptidyl peptidase 4 (DPP4), a regulator of glucose metabolism (Mourad et al., 2021), showed protective effects in several inflammatory conditions, including cardiovascular diseases, renal disorders, liver diseases, multiple sclerosis, and IBDs (Seong et al., 2019; Ning et al., 2020; Daza-Arnedo et al., 2021; Chen et al., 2022; Sharma et al., 2022). Mechanisms behind the protective effects of DPP4 inhibitors are suggested to be mediated by the suppression of oxidative stress, inflammation, apoptosis, and fibrosis in addition to enhancing autophagy (Kanasaki et al., 2014; Murase et al., 2015a; Nagamine et al., 2017; Bigagli et al., 2020; Smelcerovic et al., 2020).

BMS-477118, also known as saxagliptin, is a reversible inhibitor of DPP4 (Thareja et al., 2010). BMS-477118 is a well-tolerated antidiabetic agent. It demonstrates negligible adverse effects such as hypoglycemia, cardiovascular events, and weight gain compared with other antidiabetic medications (Yin et al., 2022). Daily treatment with BMS-477118 resulted in significant declines in serum levels of IL-1β, IL-6, IL-18, tumor necrosis factor-alpha (TNF-α), and C-reactive protein (CRP) in both preclinical and clinical studies (Birnbaum et al., 2016; Njerve et al., 2017). It also diminished the activation of the NOD-like receptor family pyrin domain containing 3 (NLRP3) inflammasome and caspase-1 activity (Birnbaum et al., 2016). Moreover, BMS-477118 treatment was associated with nitric oxide (NO) release and increased endothelial nitric oxide synthase (eNOS) activity, as well as lower circulating intercellular adhesion molecule-1 (ICAM-1) (Mason et al., 2012). Furthermore, it repressed inflammation and fibrosis by lowering transforming growth factor beta (TGF-β), monocyte chemoattractant protein 1 (MCP-1), and plasminogen activator inhibitor-1 (PAI-1) (Uchii et al., 2016).

It is important to mention that BMS-477118 has not been investigated in chronic UC. Thus, its potential benefits for this condition with unmet therapeutic needs remain unexplored. To investigate this, we examined BMS-477118s anti-inflammatory role in mitigating dextran sodium sulfate (DSS)-induced chronic UC in rats. This investigation extended to comprehend the anti-inflammatory effects via its impact on modulating the AMPK/nicotinamide adenine dinucleotide (NAD^+^)-dependent deacetylase SIRT1/Forkhead box class O 3a (FOXO3a) signaling pathway.

BMS-477118 is noteworthy for its minimal risk of hypoglycemia (Kania et al., 2011). Studies have shown that BMS-477118 at 10 mg/kg/day for 1 week did not induce hypoglycemia in normoglycemic rats (Arab et al., 2021). Additionally, our preliminary studies confirmed the absence of a hypoglycemic effect in normoglycemic rats treated with BMS-477118 at 5 mg/kg/day for 3 weeks. Our study employed the widely used DSS model of chronic UC due to its reproducibility, ease of administration, and controllability of disease progression (Chassaing et al., 2014). The pathological damage caused by daily DSS administration closely resembles the clinical and histological features of human UC, enhancing its relevance (Chassaing et al., 2014; Katsandegwaza et al., 2022). Research indicates that DSS forms nano complexes with medium-chain-length fatty acids in the lumen of colons. These nanovesicles primarily affect the distal colon and fuse with colonocyte membranes (Laroui et al., 2012). This process leads to damage to the epithelial cell monolayer lining the colon.

## 2 Materials and methods

### 2.1 Animals

In this research, male adult Sprague-Dawley rats weighing between 180 and 220 g were randomly selected and utilized. These rats were sourced from the National Institute of Cancer and given a 2-week acclimation period in a controlled environment. Throughout both the acclimation and experimental phases, the rats were kept under standard conditions and were provided with regular rodent chow and had unrestricted access to water. All procedures, including housing, care, euthanasia, and tissue collection, were conducted in accordance with the protocols approved by Delta University’s Research Ethics Committee (approval number: FPDU24120,5), ensuring adherence to ethical and legal guidelines for animal research. The committee-approved group sizes were intended to provide a more accurate estimation of the true survival rate. Additionally, a *post hoc* power analysis was run using G*power 3.1.9.7, which considered the α level, sample size, and effect size. The analysis indicated a calculated power (1-β) of 0.94.

#### 2.1.1 Experimental design

Animals were randomly categorized into distinct groups: CTRL (n = 8) served as the control group receiving no treatment; BMS 10 (n = 8) received oral BMS-477118 at 10 mg/kg as the drug control group; ch.colitis (n = 15) rats were induced with DSS to develop colitis; ch.colitis/BMS 5 (n = 15) were induced with DSS and concurrently treated with oral BMS-477118 at 5 mg/kg; and ch.colitis/BMS 10 (n = 15) were induced with DSS and treated with oral BMS-477118 at 10 mg/kg. This stratification allowed for the assessment of the effects of BMS-477118 at different doses in the context of DSS-induced colitis (Table 1). Following the previously described procedure (Hoffmann et al., 2003), chronic colitis was induced in rats through a multi-stage procedure. Initially, rats were subjected to 2% DSS administration for 7 days, followed by 10 days of treatment with 1% DSS, and finally, another 7 days of 2% DSS treatment. Throughout the experiment, diligent daily monitoring of the animals was conducted to detect any changes in their weights. Body weight loss was determined by calculating the % difference between the initial weight and the subsequent measurements. Additionally, careful monitoring of the rats’ water intake was performed to guarantee uniform exposure to DSS among all groups. The dosage of BMS-477118 was chosen based on prior studies (Dave, 2011; Mostafa et al., 2021a; Tekin et al., 2022; Othman et al., 2023). BMS-477118 was obtained from Mash Premiere Pharmaceutical Company (New Cairo, Egypt).

**TABLE 1:** Experimental design.

Exp. Groups	Day 1 – day 24
CTRL (n = 8)	—
BMS 10 (n = 8)	BMS-477118 at 10 mg/kg
ch.colitis (n = 15)	DSS
ch.colitis/BMS 5 (n = 15)	DSS/BMS-477118 at 5 mg/kg
ch.colitis/BMS 10 (n = 15)	DSS/BMS-477118 at 10 mg/kg

#### 2.1.2 Sample size justification

The DSS-treated groups were designed with 15 animals each to ensure a robust survival analysis and to generate accurate survival data. Given the significant mortality associated with DSS-induced colitis models, we selected this group size to avoid repeating the experiment in the event of substantial animal loss, thereby adhering to ethical standards by minimizing overall animal use. Mortality projections and ethical considerations were central to the research ethics committee’s approval of this sample size.

### 2.2 Percentage change in body weight

The animals’ weight was recorded daily to track any changes. The formula used to calculate the % change in body weight on day 24 was [animal’s weight at the end of day 24/animal’s weight on day 1] × 100.

### 2.3 Sample processing

Rats were euthanized by decapitation after anesthesia induction using a mixture of 12.5 mg/kg Xylazine and 87.5 mg/kg Ketamine. After dissection, the colons were measured for length and weight. They were then dissected, cleansed with cold saline, and patted dry with towels. The colons were split into sections, including distal colon specimens. One portion was stored for 24 h in 10% neutral-buffered formalin for histopathological and immunohistochemical analysis. The remaining portions were promptly cryopreserved and stored at −80°C for colorimetric, ELISA, and qRT-PCR analysis.

### 2.4 Histological examination

To achieve total dehydration, colonic tissues were removed from a neutral-buffered formalin solution and submerged in successively diluted alcohol solutions. Then, the tissues were exposed to xylene and fixed in paraffin at 56°C. Afterward, a microtome was used to section the tissue blocks encased in paraffin, creating 4–5 μm slices. The specimens were deparaffinized, rehydrated, and hematoxylin and eosin (H&E) or Masson’s trichrome stained for histological and fibrosis investigation. A blind histologist performed standard histology techniques, inspecting the specimens (20 different HPFs) with a light microscope (Olympus CX23, Tokyo, Japan). The application of the microscopic criteria of the histological grading system of inflammation was followed as described in Table 2. For fibrosis scoring, the criteria structured in Table 3 were applied.

**TABLE 2:** The microscopic criteria of the histological grading system of inflammation.

Score	Features
1	Inflammation or ulceration is limited to the mucosa layer
2	Inflammation or ulceration extends to both the mucosa and submucosa layers
3	Inflammation or ulceration involves the muscularis propria layer
4	Inflammation extends through the entire wall of the colon, including the serosa, with focal areas of ulceration
5	Widespread ulceration and inflammation that affect all layers of the colon wall, including the serosa
6	Perforation of the colon wall with inflammation and ulceration affecting all layers

**TABLE 3:** Criteria for fibrosis scoring.

Score	Features
1	No fibrosis
2	Mild fibrosis that is limited to the mucosa
3	Moderate fibrosis, extending into submucosa
4	Severe fibrosis, involving both the mucosa and submucosa extensively

### 2.5 Immunohistochemical evaluation of alpha-smooth muscle actin (α-SMA)

Immunohistochemical targeting of α-SMA was conducted using antibodies sourced from Thermo Fisher Scientific (Rockford, IL, United States). The α-SMA tissue expression was quantified by determining the % of the positive area relative to the total area. This quantification was performed using ImageJ 1.53f software (NIH, Bethesda, MD, United States).

### 2.6 Determination of the weight-to-length colon ratio

A common methodology to assess the severity of colitis progression is to quantify the colonic weight-to-length ratio. In this technique, the entire colon was measured in grams of tissue per centimeter of colonic length.

### 2.7 Assessment of the disease activity index (DAI)

The DAI is a crucial metric for assessing the severity of colitis. It offers a numerical evaluation of the presence of bloody stool, changes in body weight, and other symptoms related to chronic UC. The DAI was assessed by a gastroenterologist who was blinded on the 24th day of the trial. The sum of the scores for bloody stool, diarrhea, and percentage of body weight loss was used to calculate each rat’s DAI. The criteria for evaluating the disease severity of colitis are structured in Table 4. These criteria ensure a clear and consistent evaluation of the severity of colitis based on observable symptoms and changes in body weight.

**TABLE 4:** The criteria for evaluating the disease severity of colitis.

Parameter	Score	Features
Diarrhea	0	for normal stool
	1	for soft stool
	2	for very soft stool
	3	for watery diarrhea
Weight loss	0	for no loss
	1	for 1%–5% loss
	2	for 6%–10% loss
	3	for 11%–15% loss
	4	for 16%–20% loss
Bloody stool	0	for no hemoccult
	1	for hemoccult-positive
	2	for traces of blood
	3	for severe rectal bleeding

### 2.8 Assessment of the macroscopical damage index (MDI)

A blind pathologist examined the macroscopical features of tissue damage. The scoring system used to evaluate each rat’s MDI is structured in Table 5. This system provides a comprehensive and graduated assessment of colonic damage, from mild hyperemia to extensive ulceration and inflammation.

**TABLE 5:** The criteria for evaluating the macroscopical damage in colitis.

Score	Features
0	no damage
1	hyperemia without ulcers
2	a linear ulcer without significant inflammation
3	a linear ulcer with inflammation at one site
4	two or more sites with inflammation or ulceration
5	two or more major sites of inflammation or ulceration or one site with inflammation or ulceration extending more than or equal to 1 cm along the length of the colon
6-10	damage covering more than or equal to 2 cm along the colon, with the score increasing by one for each additional centimeter of involvement

### 2.9 Assessment of zonula occludens-1 (ZO-1) and occludin (OCLN)

The ZO-1 and OCLN levels were determined in tissue homogenate using sandwich ELISA following instructions given by CUSABIO (Wuhan, China). Colon tissue samples weighing 100 mg each were rinsed with cold PBS and homogenized in 1 mL of the same buffer. Following centrifugation at 5000 *g* for 5 min, the resulting supernatant was collected and utilized for subsequent assays. Each assay was carried out in duplicate.

### 2.10 Determination of reactive oxygen species (ROS), malondialdehyde (MDA), reduced glutathione (GSH), and superoxide dismutase (SOD)

To detect ROS in tissues, 200 mg of samples were homogenized in a chilled Tris-HCl (40 mM, pH 7.4) at a ratio of 1:10 w/v. 100 μL of the homogenates was combined with 1 mL of Tris-HCl, and then 10 μM of 2′, 7′-dichlorofluorescein diacetate was added. Following this, the mixture was incubated for 30 min at 37°C. After incubation, the FI was measured in a microplate reader with excitation at 485 nm and emission at 525 nm. For the levels of MDA, GSH, and SOD, we used Bio-diagnostic kits (Giza, Egypt) and followed the manufacturer’s instructions. All oxidative stress marker tests were carried out in duplicate.

### 2.11 Assessment of myeloperoxidase (MPO) activity

Colon tissues were initially washed in cold PBS, followed by homogenization in an assay buffer (Abcam). After centrifugation at 13,000 × g for 10 min at a low temperature, the resulting supernatants were carefully transferred to sterile tubes. Standard or sample solutions were then dispensed in triplicate into separate wells, adjusting each to 50 µL with the buffer. A 50 µL reaction mix was added to each well and allowed to incubate at 25°C for 30 min. Subsequently, 2 µL of a stop mix was introduced, followed by an additional 10-min incubation at room temperature. Next, 50 µL of TNB was added, thoroughly mixed, and incubated for a further 10 min at room temperature. Finally, readings were taken at 412 nm. One unit of MPO activity was defined as the amount able to hydrolyze the substrate to generate taurine chloramine, consuming 1.0 μmol of TNB per minute at 25°C.

### 2.12 Analysis of cytokine levels: TNF-α, IL-6, IL-10, IL-4, and TGF-β

TNF-α and IL-10 levels were measured using ELISA kits provided by LifeSpan BioSciences, Inc. (Seattle, WA, United States). R&D System (Minneapolis, MN, United States) kits were used to assess IL-6 and IL-4 levels, whereas an eBioscience kit (Vienna, Austria) was used to quantify TGF-β levels. All assays were carried out in exact accordance with the manufacturer’s instructions. Each cytokine assay was repeated twice.

### 2.13 Determination of FOXO3a (nuclear and cytosolic fractions)

Colon tissues were weighed, then sectioned into minute fragments, washed with PBS, and placed in a homogenizer. After adding a cytoplasmic extraction buffer containing phenylmethanesulfonyl fluoride (PMSF) (1 mM) and a protease inhibitor cocktail, the tissue fragments were homogenized with 50–60 strokes. The homogenate was then incubated on ice for 15 min before being centrifuged at 14,000 × g at 4°C for 10 min. The supernatant representing the cytosolic fraction was carefully collected. The protein concentration in this fraction was measured using the Bradford reagent. The nuclear pellet was resuspended twice in the nuclear wash buffer and centrifuged at 3000 *g* for 4 min. The supernatant was discarded each time. The pellet was then resuspended in a nuclear extraction solution containing 1 mM of PMSF and a protease inhibitor cocktail. The tubes were incubated on ice for 15 min, with a 5-s vortex every 3 minutes. For effective extraction of nuclear proteins, the extract was sonicated three times for 10 s each. The tubes were then centrifuged at 18,000 × g for 10 min at 4°C, and the supernatant, including a nuclear extract, was collected. The protein concentration of the nuclear extract was finally determined using a Bradford reagent. The presence of FOXO3a in cytosolic or nuclear extracts was detected using the DNA-binding indirect ELISA method. In this immunoassay, streptavidin was immobilized on the assay plate, and particular biotinylated double-stranded oligonucleotides were coupled to it via a high-affinity biotin-streptavidin interaction. This was followed by inhibiting any superfluous binding sites within each well before introducing the sample containing the target FOXO3a. Primary antibodies were used to precisely bind to activated FOXO3a, which was already coupled to the immobilized dsDNA oligonucleotide (LSBio) on the plate-coated streptavidin. An HRP-conjugated secondary antibody specific for rabbit IgGs was then introduced, allowing for selective binding to the primary antibody and subsequent colorimetric detection upon the addition of the TMB substrate. A blue TMB di-imine product was produced, the intensity of which was proportional to the dsDNA binding activity of FOXO3a in the sample. A stop solution was added, and the absorbance was subsequently measured using a spectrophotometer at 450 nm. Each assay was performed in duplicate.

### 2.14 Determination of AMPK activity

After harvest, tissues were washed with a cold PBS buffer (0.01M, pH = 7.4) to eliminate any leftover blood. The tissue was then weighed, chopped into small pieces, and homogenized in PBS with a cocktail of protease and phosphatase inhibitors. This homogenization was performed with a cooled homogenizer, and the suspension was further disrupted by sonication. Following these processes, the homogenates were centrifuged for 5 min at 5000 × g to separate the supernatant. The total protein concentration was then measured with a BCA reagent. The experiment was carried out on a 96-well plate pre-coated with a capture antibody. p-AMPK was identified with a biotin-conjugated antibody (Fine Test, Wuhan Fine Biotech Corp., China). An HRP-Streptavidin conjugate was used, and the HRP enzymatic reaction was observed using TMB substrates, yielding a blue product that became yellow when the stop solution was added. The intensity of the yellow color was related to the amount of p-AMPK caught on the plate. The absorbance was measured at 450 nm. The experiment was repeated two times.

### 2.15 Determination of nuclear factor kappa B (NFĸB) activity

The colon tissue samples were first weighed and then dissected into small fragments, which were subsequently placed in a homogenizer. Following the addition of 5 mL of pre-extraction buffer (Abcam), which included 5 µL of DTT solution for each Gram of tissue, the tissue fragments were homogenized with 50–60 strokes. The homogenate was incubated on ice for 15 min and then centrifuged at 14,000 × g at 4°C for 10 min. The supernatant was collected carefully. The extraction buffer was modified by adding a DTT solution, a protease inhibitor, and a phosphatase inhibitor cocktail. This modified extraction buffer was then added to the nuclear pellet. The extract was incubated on ice for 15 min, with a 5-s vortex occurring every 3 min. To enhance the extraction of nuclear proteins, the extract was sonicated three times for 10 s each. The suspension was then centrifuged at 18,000 × g at 4°C for 10 min, and the resulting supernatant was transferred into a fresh microcentrifuge vial. The protein concentration of the nuclear extract was finally measured using a Bradford reagent. In the NFĸB p65 assay, a dsDNA sequence, which included the NFĸB response element, was immobilized on the base of 96-well plate wells. The NFĸB present in the nuclear extract is bound to the NFĸB response element, and this interaction was subsequently detected with an anti-NFĸB p65 antibody. A secondary antibody coupled to HRP was then introduced, enabling the detection and quantification of NFĸB p65 activity at 450 nm. The assay was performed twice for accuracy.

### 2.16 Determination of SIRT1 expression and activity levels

Homogenization of tissue was performed on ice using a glass homogenizer, with 1 mL of lysis buffer containing 1 mM PMSF for every 50 mg of colon tissue sample. The resulting suspension was sonicated, and the lysate was centrifuged at 10,000 × g for 5 min. The supernatant was then collected for the assay using a kit from Cloud-Clone Corp. (Houston, TX, United States). Furthermore, intracellular SIRT1 activity was measured in the prepared immunoprecipitate using an anti-SIRT1 antibody from Abcam. The tissue lysis buffer comprised 20 mM Tris-HCl (pH 7.5), 250 mM NaCl, 1 mM EDTA, 1 mM EGTA, 1% Triton X-100, and 1 mM DTT. Following centrifugation at 13,000 g for 10 min at 4°C, the supernatant was discarded. Nuclear protein was then collected by centrifugation at 20,000 g for 10 min after sonication. Protein concentration was determined using the BCA protein assay. Deacetylase activity was measured by measuring this fluorescence intensity after mixing with a fluorescence-labeled acetylated peptide substrate using a Tecan SpectraFluor Plus microplate reader (λ excitation = 340 nm and λ emission = 440 nm) (Huang et al., 2019; Wang et al., 2019). Each assay was conducted in duplicate.

### 2.17 Determination of UNC-51-like autophagy activating kinase 1 (ULK1) and Beclin 1

The colon tissues were cleaned with cold PBS buffer (0.01 M, pH = 7.4) on ice to remove any remaining blood. Then, the tissues were homogenized using 9 mL of PBS per 1 g of tissue and 1 mM of freshly prepared PMSF as a protease inhibitor, following the manufacturer’s instructions. After homogenization, the samples were sonicated in chilled conditions and then centrifuged at 5000 *g* for 5 min. Protein levels were determined using the bicinchoninic acid protein assay. Beclin 1 and ULK1 were assayed using the Fine Test and MyBioSource (San Diego, CA, United States) assays, respectively, as per the manufacturer’s instructions, with each assay conducted in duplicate.

### 2.18 Determination of p62

Before the homogenization process, the tissues were sliced into tiny fragments, then rinsed thoroughly in ice-cold PBS to remove excess blood, and weighed precisely. The tissues were then homogenized using Cloud-Clone Corp’s freshly produced lysis solution with 1 mM PMSF. The homogenization process involved using 1 mL of lysis buffer for every 50 mg of tissue sample, and it was carried out on ice with a homogenizer. The suspension was sonicated until clear. After sonication, the lysate was centrifuged at 10,000 × g for 5 min, and the supernatants were collected for the test. The assay was performed using the MyBioSource ubiquitin-binding protein p62 assay following the manufacturer’s instructions, and each assay was conducted in duplicate.

### 2.19 Determination of BCL2-associated X protein (Bax) and B-cell lymphoma 2 (BCL-2) levels

100 mg of colon tissue was cleaned with PBS and homogenized in 1 mL of the same buffer. The homogenates were centrifuged for 5 min at 5000 × g, 4°C. The supernatant was removed and assayed for Bax using an ELISA kit from Biovision Inc. (CA, United States). The concentration of BCL-2 was determined using a CUSABIO ELISA test kit. Each protocol followed the corresponding manufacturer’s instructions, and each assay was carried out in duplicate.

### 2.20 Determination of active caspase-3

We measured the levels of active caspase-3 using a kit from MyBioSource, which included a polyclonal antibody specific for active caspase-3 and an HRP-conjugated active caspase-3. The observed color intensity was inversely related to the amount of active caspase-3 in the samples. The antibody used had a limited number of binding sites, causing a competition between the active caspase-3 in the samples and the HRP-conjugated active caspase-3. Consequently, as more binding sites were taken up by the active caspase-3 from the samples, fewer sites were available for the HRP-conjugated version to bind. For accuracy, we conducted the measurement twice.

### 2.21 Statistical analysis

The statistical evaluation was performed using GraphPad Prism version 10 (GraphPad Software Inc., La Jolla, CA, United States). Results were presented as the mean ± standard deviation (SD). Differences between groups were identified using one-way ANOVA, followed by Tukey’s kramar *post hoc* test. All statistical tests were deemed significant at a *p*-value of less than 0.05. Additionally, a *post hoc* power analysis was carried out using G*Power 3.1.9.7.

## 3 Results

### 3.1 Effects of BMS-477118 on histopathological outcomes, fibrosis, and α-SMA immunoexpression in rats with UC

**FIGURE 1:**
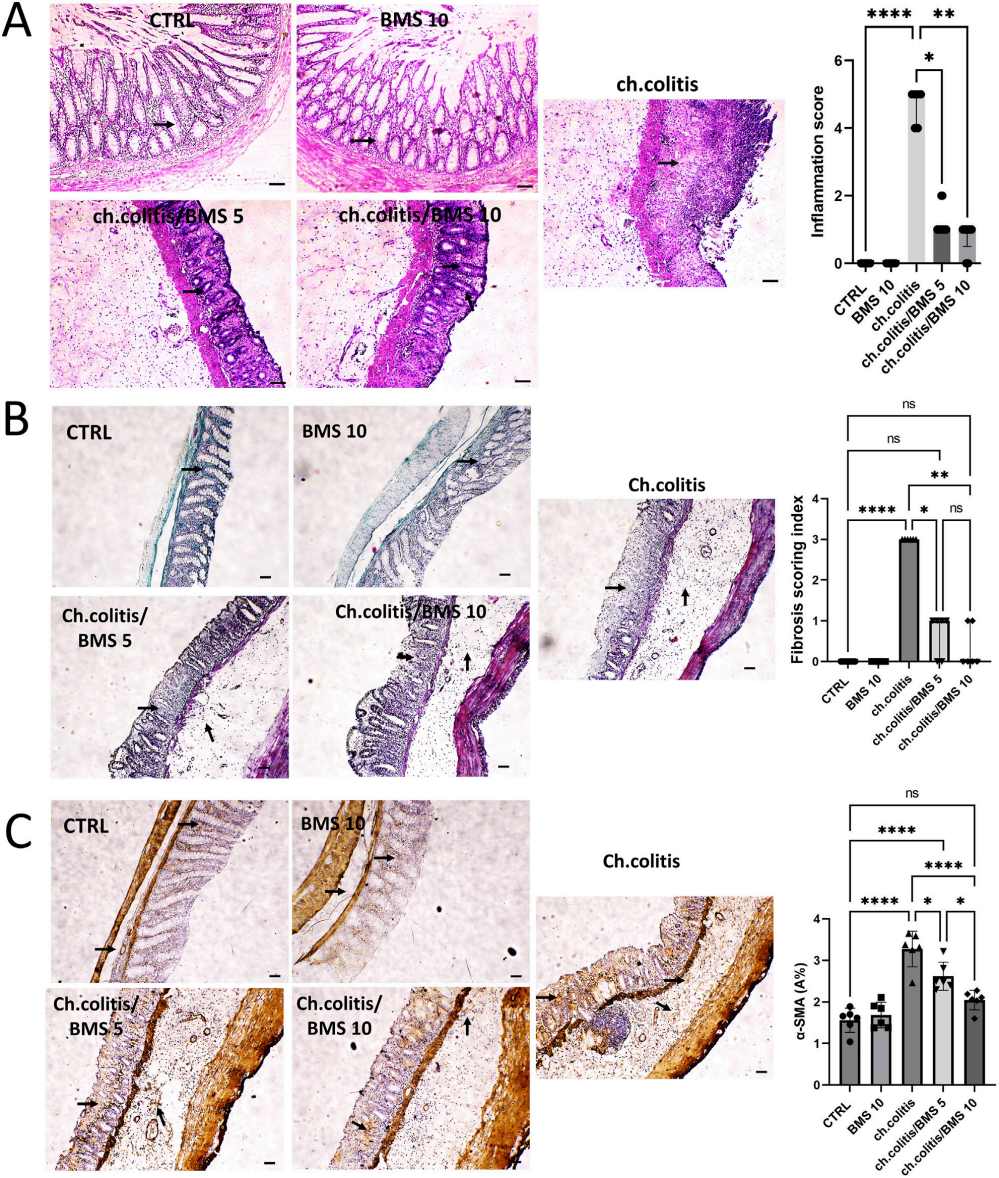
Effect of BMS-477118 on histological changes, fibrotic tissue deposition, and α-SMA immunoexpression in rats with chronic UC. **(A)** Photomicrographs from the CTRL and BMS 10 groups exhibit normal mucosal epithelium, crypts, and goblet cells (arrows). In contrast, the ch.colitis group shows significant epithelial damage, including extensive crypt loss, detachment of epithelial cells from the lamina propria, inflammatory cell infiltration, subepithelial fibrosis, thickening of the lamina propria, and widespread edema. The ch.colitis/BMS 5 group demonstrates moderate crypt regeneration (arrows), while the ch.colitis/BMS 10 group displays marked crypt regeneration (arrows). The inflammation scores are significantly higher in rats with chronic colitis compared to the CTRL group, and treatment with BMS-477118 at both low and high doses significantly reduced these scores in the treated groups compared to the untreated rats with chronic UC. H&E stain, ×100. Scale bar = 50 µm. Data are presented as median ± IQR (n = 6). Statistical analysis was performed using Kruskal–Wallis’ test, followed by Dunn’s *post hoc* test. **(B)** A photomicrograph of colon sections shows increased fibrotic tissue deposition in the mucosa and submucosa of the ch.colitis group compared to the healthy CTRL group, as indicated by arrows pointing to the light green staining, which marks collagen deposition and distribution. Both low and high doses of BMS-477118 reduced fibrotic tissue deposition compared to the ch.colitis group. The fibrosis scoring index is significantly lower in treated groups, confirming these histological findings. Masson’s trichrome stain, ×100. Scale bar = 50 µm. Data are presented as median ± IQR. Statistical analysis was performed using Kruskal–Wallis’ test, followed by Dunn’s *post hoc* test. **(C)** A photomicrograph of colon sections from the untreated ch.colitis group shows increased α-SMA immunoexpression in the mucosa and submucosa compared to the CTRL group, as indicated by arrows pointing to the brown staining. Treatment with BMS-477118 at both low and high doses reduced α-SMA immunoexpression compared to the ch.colitis group. The α-SMA A% data confirm these findings, with statistically significant differences among the groups. α-SMA IHC, ×100. Scale bar = 50 µm. Data are presented as mean ± SD (n = 6). Statistical analysis was performed using one-way ANOVA followed by Tukey’s *post hoc* test. *, *p* < 0.05; **, *p* < 0.01; ***, *p* < 0.001; ****, *p* < 0.0001; ns = not significant.

Photomicrographs from the control group (CTRL) and the BMS 10 group showed normal mucosal epithelium, crypts, and goblet cells, while the chronic colitis group exhibited significant epithelial damage. This damage was characterized by extensive crypt loss, detachment of epithelial cells from the lamina propria, inflammatory cell infiltration, subepithelial fibrosis, thickening of the lamina propria, and widespread edema. Treatment with BMS-477118 at a low dose (BMS 5) led to moderate crypt regeneration, whereas the high dose (BMS 10) resulted in marked crypt regeneration, indicating a substantial reparative effect at the higher dose. Correspondingly, rats with chronic colitis showed higher inflammation scores compared to the control group, but BMS-477118 administration at both low and high doses significantly reduced these inflammation scores compared to untreated rats with chronic UC (Figure 1A). as depicted in Figure 1B, untreated rats with chronic UC (ch. colitis group) had increased fibrotic tissue deposition in the mucosa and submucosa compared to healthy control rats. This increase was evidenced by light green staining, which indicated the extent of collagen deposition and distribution. Treatment with both low and high doses of BMS-477118 resulted in a reduction in fibrotic tissue deposition relative to the chronic colitis group. Statistical comparisons of the fibrosis scoring index among the experimental groups confirmed these histological findings, supporting the antifibrotic effect of BMS-477118. Figure 1C illustrates that the untreated rats from the chronic colitis group showed an increase in α-SMA immunoexpression in the mucosa and submucosa compared to the healthy control group. This increase was marked by brown staining. Treatment with both low and high doses of BMS-477118 significantly reduced α-SMA immunoexpression compared to the chronic colitis group. Statistical comparisons of α-SMA A% across the different experimental groups confirmed these immunohistochemical results, indicating that BMS-477118 effectively reduces markers of smooth muscle activation associated with fibrotic processes.

### 3.2 Effects of BMS-477118 on colonic parameters, survival, body weight, and tight junction proteins in rats with chronic UC

**FIGURE 2:**
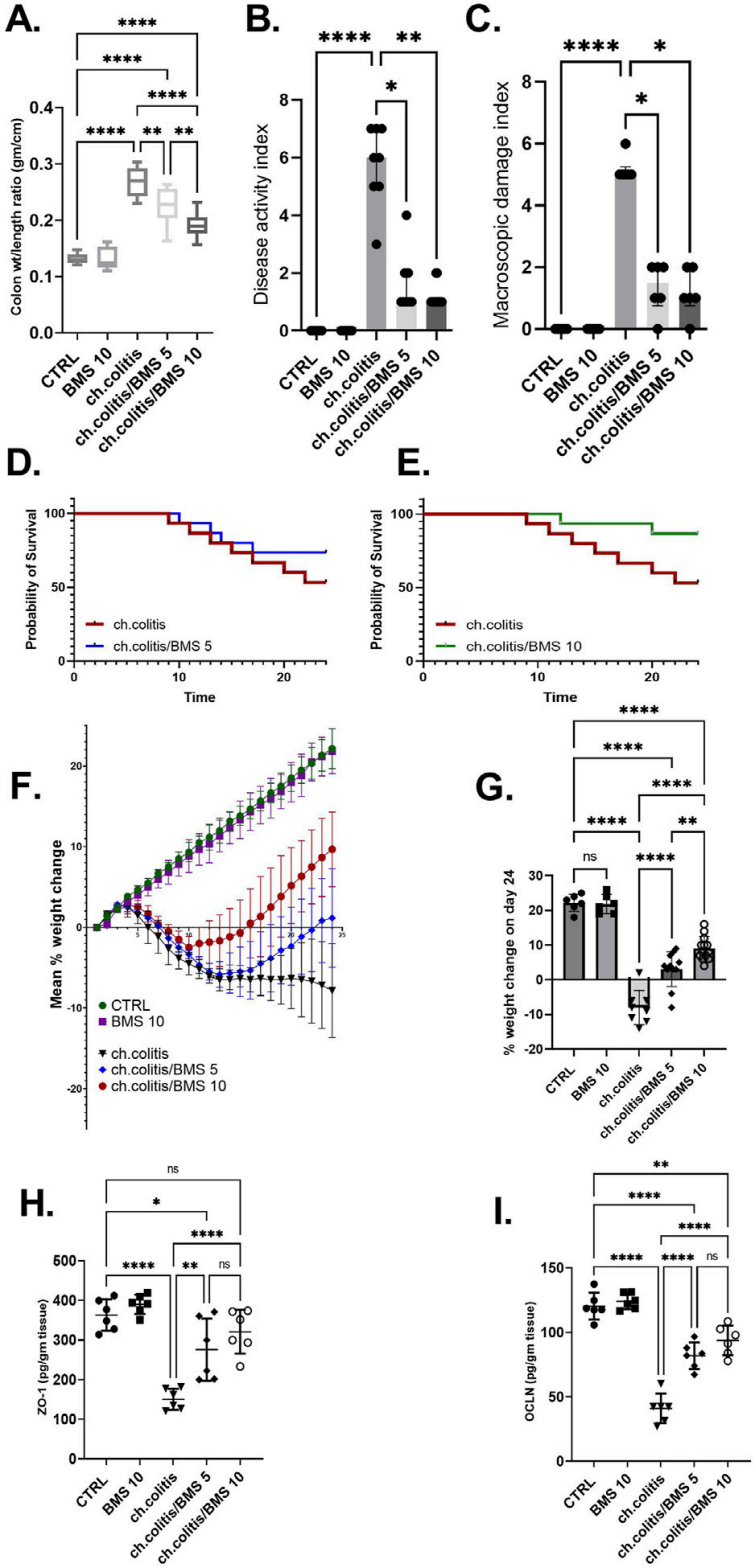
Effects of BMS-477118 on colon parameters, survival, body weight, and tight junction protein levels in rats with chronic UC. **(A–C)** Effect of BMS-477118 on the colon weight-to-length ratio **(A)**, Disease Activity Index (DAI) **(B)**, and Macroscopic Damage Index (MDI) **(C)**. Data for the colon weight-to-length ratio are presented as mean ± SD, and statistical analysis was performed using one-way ANOVA followed by Tukey’s *post hoc* test. Data for DAI and MDI are presented as median ± IQR (n = 6–13), and statistical analysis was performed using the Kruskal–Wallis test followed by Dunn’s *post hoc* test. **(D)** Survival probability in the ch.colitis group compared to the ch.colitis/BMS 5 group. **(E)** Survival probability in the ch.colitis group compared to the ch.colitis/BMS 10 group. Data were analyzed using the Log-rank (Mantel-Cox) test, with each step down in the curve representing an animal death in that group. 15 rats per group were included for each observation. **(F)** Graphical representation of body weight change throughout the experiment. **(G)** Final percentage change in body weight on day 24. **(H, I)** Effect of BMS-477118 on the levels of tight junction proteins ZO-1 **(H)** and OCLN **(I)**. Data are presented as mean ± SD (n = 6). Statistical analysis was performed using one-way ANOVA followed by Tukey’s *post hoc* test. *, *p* < 0.05; **, *p* < 0.01; ****, *p* < 0.0001; ns = not significant.

Long-term administration of DSS in rats resulted in a significant increase in the colonic weight-to-length ratio, DAI, and MDI compared to healthy rats. However, treatment with BMS-477118 at doses of 5 mg/kg or 10 mg/kg led to significant reductions in these parameters compared to the untreated chronic colitis group (Figures 2A–C, respectively). DSS exposure significantly decreased the survival probability of rats. While treatment with a low dose of BMS-477118 (5 mg/kg) did not result in a significant improvement in survival compared to untreated rats with DSS-induced colitis (*p*-value = 0.3035), a higher dose of 10 mg/kg significantly improved survival probability compared to the untreated chronic colitis group (*p*-value = 0.0484) (Figures 2D,E, respectively). Moreover, the chronic colitis group exhibited a significantly greater percentage of weight loss compared to the CTRL group, as shown in Figure 2F (mean % weight change over time) and Figure 2G (final % weight change on day 24). However, both BMS-477118-treated groups (ch. colitis/BMS 5 and ch. colitis/BMS 10) demonstrated significantly lower percentages of weight loss compared to the untreated chronic colitis group. Weight change was calculated relative to the initial animal weight on the first day of the experimentation. Furthermore, chronic UC induction resulted in a significant decrease in the expression of tight junction proteins, such as ZO-1 and OCLN, compared to the control group (Figures 2H,I, respectively). This reduction suggests a disruption of the tight junctions in the colonic tissue, contributing to increased intestinal permeability and inflammation. However, administration of BMS-477118 at both 5 mg/kg and 10 mg/kg doses led to a significant upregulation of ZO-1 and OCLN expression compared to the untreated chronic colitis group.

### 3.3 BMS-477118 relieved oxidative stress in rats with chronic UC

**FIGURE 3:**
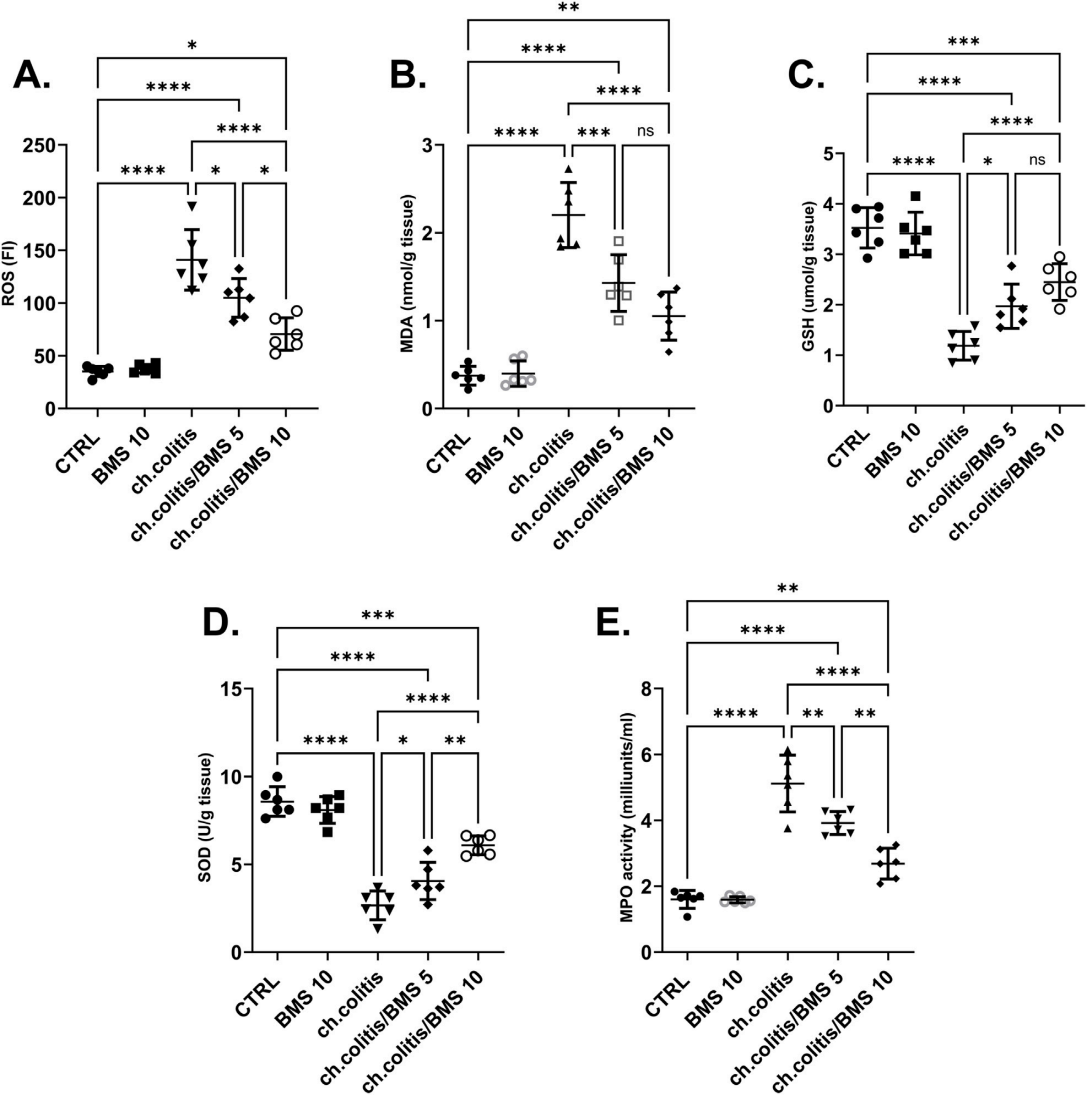
Effect of BMS-477118 on oxidative stress markers. The figure panels illustrate various biochemical markers affected by chronic administration of DSS and the subsequent treatment with BMS-477118. **(A)** ROS levels show a significant elevation due to DSS, which BMS-477118 reduces at both 5 mg/kg and 10 mg/kg doses. **(B)** MDA levels are markedly increased in the chronic colitis group and significantly declined with BMS-477118 administration. **(C)** GSH levels and **(D)** SOD levels are significantly diminished due to DSS but show significant increases following BMS-477118 treatment. **(E)** MPO activity is significantly elevated in the untreated chronic colitis group and significantly reduced in both chronic colitis groups treated with BMS-477118 (5 and 10 mg/kg). Data are presented as mean ± SD (n = 6). Statistical analysis was performed using one-way ANOVA followed by Tukey’s *post hoc* test. *p < 0.05, **p < 0.01, ***p < 0.001, ****p < 0.0001.

The colonic tissue levels of ROS were significantly elevated after chronic administration of DSS compared to the normal healthy, untreated group. However, the daily administration of BMS-477118 at 5 mg/kg or 10 mg/kg resulted in a significant reduction in ROS levels compared with untreated rats with UC, indicating an antioxidant effect of this drug (Figure 3A). Similarly, MDA levels showed a marked increase in the ch.colitis group compared to the CTRL group. Furthermore, MDA levels significantly declined after administration of either low or high doses of BMS-477118 (Figure 3B). Regarding the effect on GSH (Figure 3C) and SOD (Figure 3D), DSS led to a significant diminution in their levels compared to the CTRL levels. However, administration of BMS-477118 at doses of either 5 mg/kg or 10 mg/kg to rats with UC resulted in a significant increase in both markers and an enhancement of antioxidant activity compared with untreated rats with UC. Furthermore, the MPO activity was significantly increased in the ch.colitis group compared to the CTRL group (Figure 3E). Nevertheless, MPO activity was significantly reduced in both ch.colitis/BMS 5 and ch.colitis/BMS 10 groups compared to the ch.colitis group.

### 3.4 Effect of BMS-477118 on pro-inflammatory and anti-inflammatory cytokines

**FIGURE 4:**
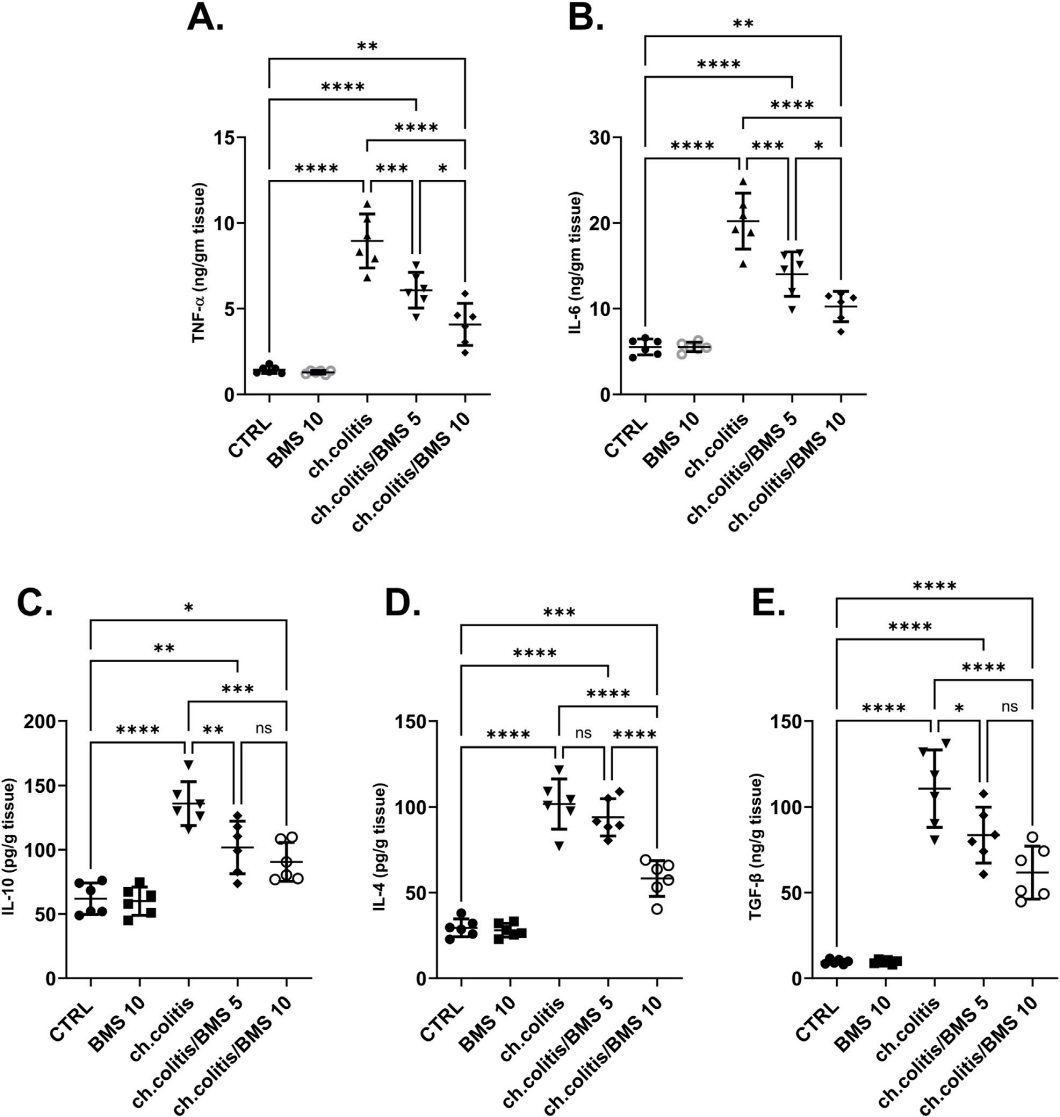
Effect of BMS-477118 on pro-inflammatory and anti-inflammatory cytokines. The graphs represent the levels of TNF-α **(A)**, IL-6 **(B)**, IL-10 **(C)**, IL-4 **(D)**, and TGF-β **(E)**. The cytokine levels were significantly elevated in the untreated UC group compared to the healthy controls. Administration of BMS-477118 at both low and high doses resulted in a significant reduction in cytokine levels. Data are presented as mean ± SD (n = 6). Statistical analysis was performed using one-way ANOVA followed by Tukey’s *post hoc* test. *p < 0.05, **p < 0.01, ***p < 0.001, ****p < 0.0001.

The levels of TNF-α and IL-6 (pro-inflammatory cytokines), along with IL-10, IL-4, and TGF-β (anti-inflammatory cytokines), were significantly elevated in the colonic tissue of rats afflicted with UC (Figures 4A–E, respectively) compared to the cytokine levels in healthy, untreated rats, indicating a clear correlation between the disease state and cytokine expression. Administration of either low or high doses of BMS-477118 resulted in a significant reduction in the levels of all these cytokines. The increase in anti-inflammatory cytokines indicates an attempt by the body to resolve chronic inflammation.

### 3.5 Molecular effects of BMS-477118 in the colons of rats with chronic UC

**FIGURE 5:**
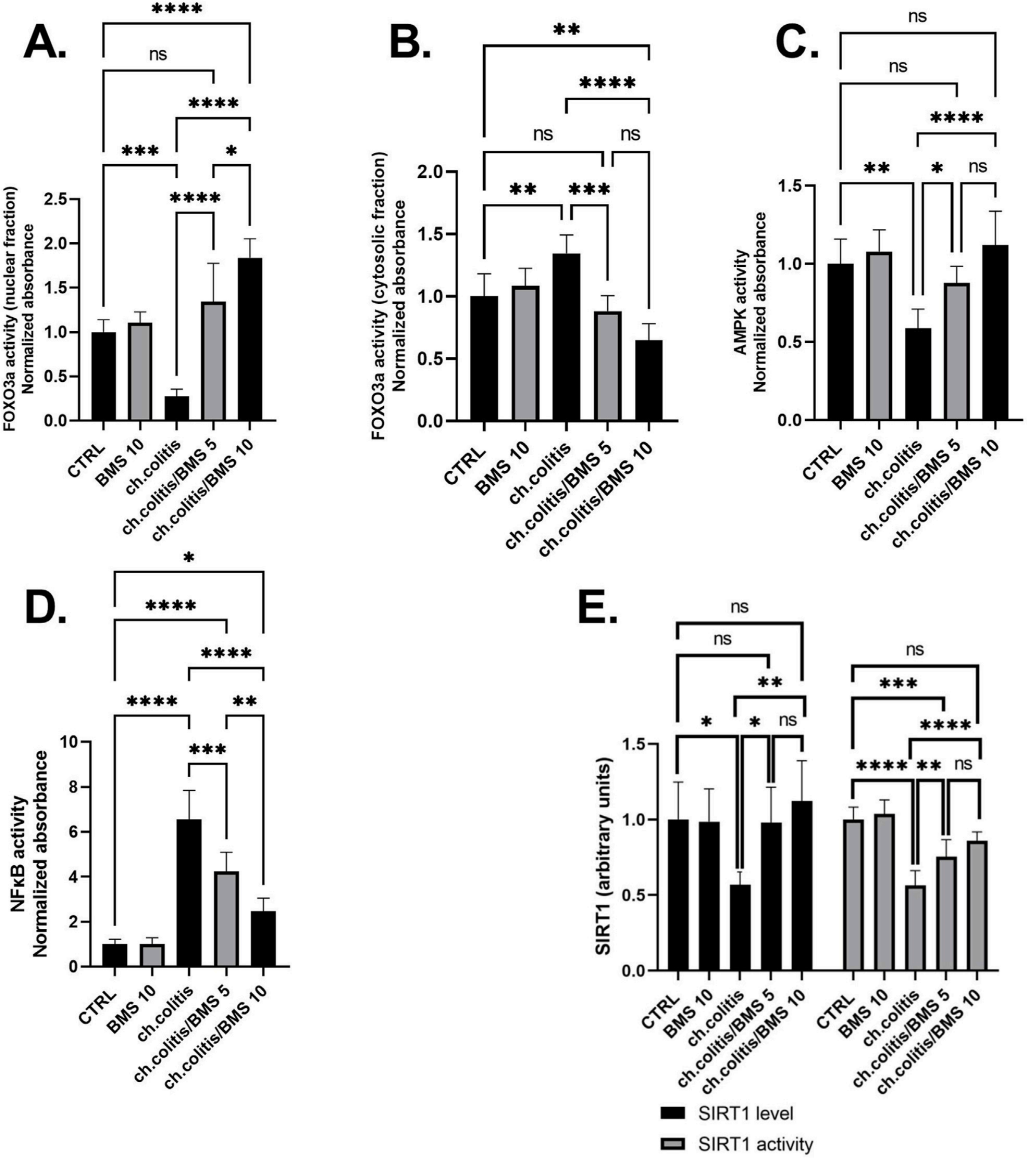
Effect of BMS-477118 on FOXO3a nuclear **(A)** and cytosolic **(B)** fractions, AMPK activity **(C)**, NFκB activity **(D)**, and SIRT1 **(E)**. Data are presented as mean ± SD (n = 6). Statistical analysis was performed using one-way ANOVA followed by Tukey’s *post hoc* test. *, *p* < 0.05; **, *p* < 0.01; ***, *p* < 0.001; ****, *p* < 0.0001.

FOXO3a is primarily localized in the nucleus when it’s active. In the nucleus, it binds to specific DNA sequences, influencing the expression of target genes involved in cell survival, metabolism, and longevity (Manoharan et al., 2023). The nuclear fraction of FOXO3a protein was significantly reduced in rats subjected to chronic doses of DSS compared to healthy, untreated rats. However, administering BMS-477118 at doses of 5 or 10 mg/kg to rats with UC counteracted the effect of DSS. This indicates the potential therapeutic impact of BMS-477118 in modulating FOXO3a activity (Figure 5A). Inactivity of FOXO3a leads to its phosphorylation. Phosphorylated FOXO3a is exported from the nucleus to the cytoplasm. In the cytoplasm, it interacts with other proteins and remains sequestered, preventing its transcriptional activity (Manoharan et al., 2023). There was a significant elevation of the cytosolic fraction of FOXO3a in the group with chronic colitis compared to the CTRL group. This elevation was mitigated upon treatment with both the low and high doses of BMS-477118 (Figure 5B).

In the group of rats with chronic colitis, a significant decrease in the activity of AMPK was observed when compared to the CTRL group. However, the administration of BMS-477118, at both low and high doses, to rats with chronic UC resulted in a significant increase in AMPK activity, as compared to the untreated rats with chronic UC (Figure 5C). Additionally, the evaluation of NFκB DNA binding activity in the untreated chronic colitis group revealed a significant increase compared to the CTRL’s NFκB activity. However, the administration of BMS-477118, at doses of either 5 mg/kg or 10 mg/kg, to rats with chronic UC resulted in a significant reduction in NFκB activity compared to the untreated chronic colitis group (Figure 5D). Furthermore, in the colonic tissues of untreated rats with chronic UC, there was a significant decrease in the expression and activity of SIRT1 compared to healthy untreated rats. However, the administration of BMS-477118 at either low or high doses to rats with chronic UC resulted in increased levels and activity of SIRT1 compared to the untreated chronic colitis group. This suggests a potential therapeutic role of BMS-477118 in modulating SIRT1 in the context of UC (Figure 5E).

### 3.6 Modulation of autophagy and apoptosis markers by BMS-477118 in rats with chronic UC

**FIGURE 6:**
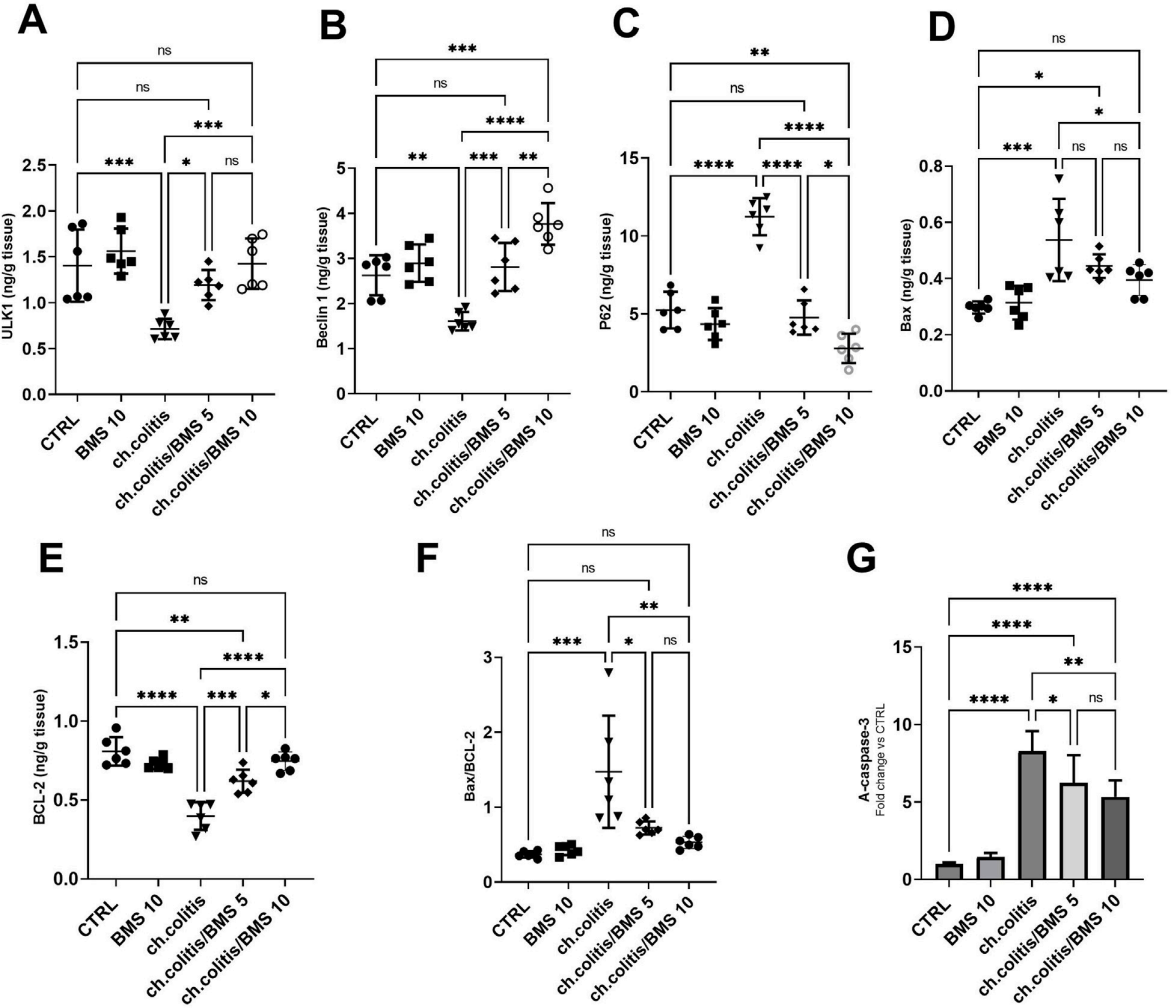
Effects of BMS-477118 on autophagy and apoptosis markers in rats with chronic UC. ULK1 **(A)**, Beclin 1 **(B)**, P62 **(C)**, Bax **(D)**, BCL-2 **(E)**, the Bax/BCL-2 ratio **(F)**, and active caspase-3 levels **(G)**. Data are presented as mean ± SD (n = 6). Statistical analysis was performed using one-way ANOVA followed by Tukey’s *post hoc* test. *, *p* < 0.05; **, *p* < 0.01; ***, *p* < 0.001; ****, *p* < 0.0001; ns = not significant.

In untreated rats with chronic colitis, there was a significant decrease in the levels of ULK1 and Beclin 1 compared with the control, healthy group. However, upon treatment with either 5 mg/kg or 10 mg/kg of BMS-477118, the levels of ULK1 and Beclin 1 in the chronic colitis group were significantly increased compared to the untreated chronic colitis group (Figures 6A,B, respectively). Additionally, there was a significant increase in the levels of p62 following the chronic induction of UC compared to healthy rats. However, the administration of 5 or 10 mg/kg of BMS-477118 to rats with chronic UC downregulated P62 levels (Figure 6C). On the other hand, the study evaluated apoptotic activity in colonic tissues by measuring the levels of Bax (Figure 6D) and BCL-2 (Figure 6E), their ratio (Figure 6F), and the cleaved caspase-3 (Figure 6G). We revealed that the ratio of Bax/BCL-2 and A-caspase-3 levels were significantly elevated in the untreated colitis group compared to the CTRL group. Nonetheless, BMS-477118 at doses of 5 or 10 mg/kg decreased the ratio of Bax/BCL-2 and A-caspase-3 levels compared to the untreated chronic colitis group.

### 3.7 Correlation analysis of the biochemical parameters examined in rats with UC

**FIGURE 7:**
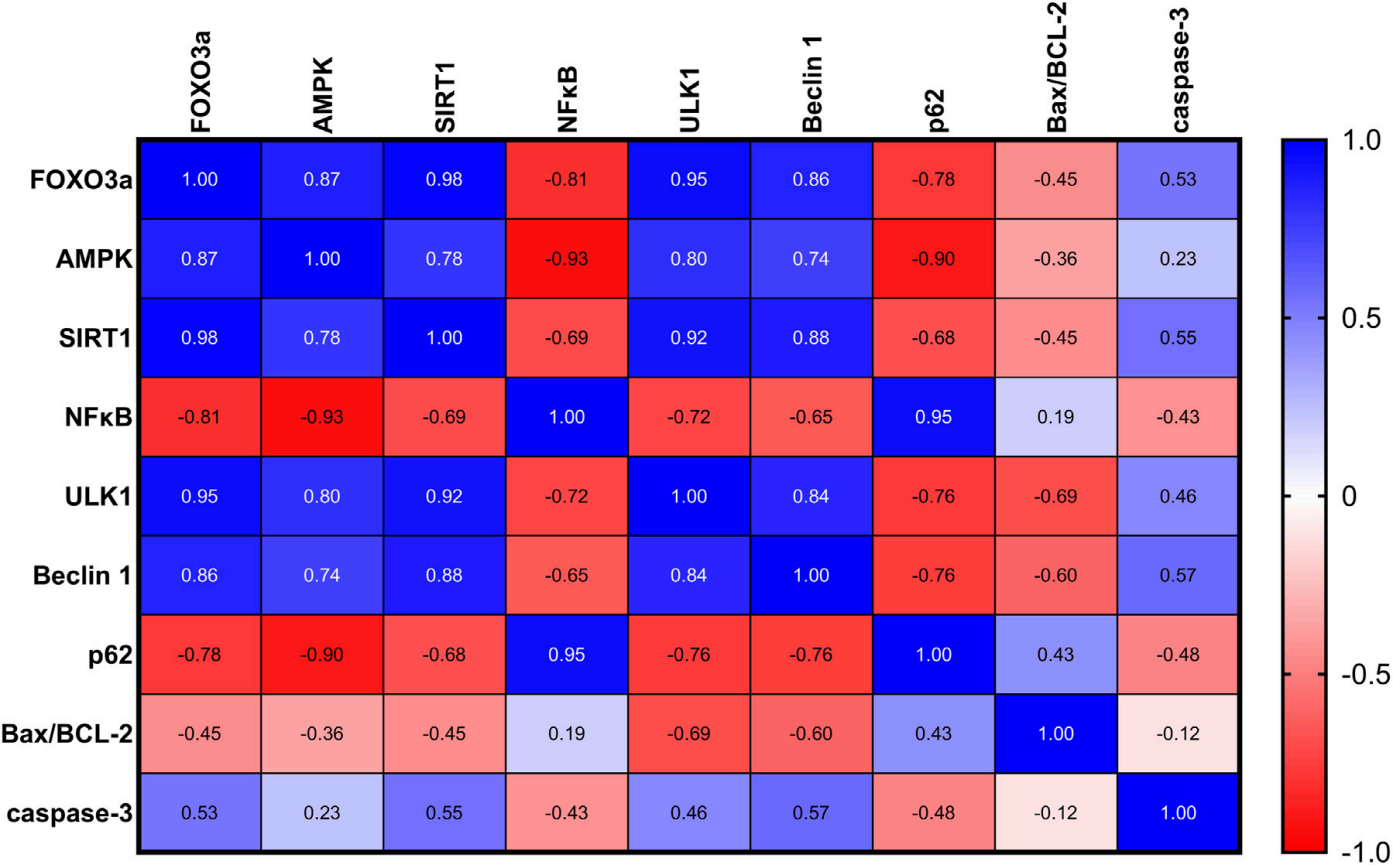
A correlation analysis of the biochemical parameters in rats with UC revealed significant positive correlations between nuclear FOXO3a and each of the measured proteins: AMPK, SIRT1, ULK1, and Beclin-1, indicating potential regulatory interactions. Additionally, significant negative correlations were found between nuclear FOXO3a and NFκB and P62, suggesting an inverse relationship. FOXO3a was found to be non-correlated with the Bax/BCL-2 ratio and A-caspase-3 in the ulcerated colon.

As depicted in Figure 7, in rats with UC, correlation analysis of biochemical parameters unveiled significant positive correlations between nuclear FOXO3a and each of the measured proteins: AMPK, SIRT1, ULK1, and Beclin-1, indicating potential regulatory interactions. Furthermore, significant negative correlations were found between nuclear FOXO3a and NFκB and P62, suggesting an inverse relationship. However, FOXO3a was found to be uncorrelated with the Bax/BCL-2 ratio and A-caspase-3 in the ulcerated colon.

## 4 Discussion

Chronic UC is a severe form of inflammatory bowel disease (IBD) marked by persistent inflammation in the colon and rectum. Despite the availability of various treatment options, many patients struggle to achieve long-term remission, highlighting a significant unmet therapeutic need. Dysregulation of AMPK activity has been implicated in major chronic disorders such as colitis. This leads to disturbances in a multitude of biological and physiological processes (Antonioli et al., 2016). An aberration in AMPK activity brings on the activation and nuclear translocation of NFκB (Xiang et al., 2019). Consequently, NFκB augments the synthesis of IL-6 and TNF-α, both of which act as proinflammatory mediators and initiate inflammatory responses (Mankan et al., 2009; Youssef et al., 2022; Zohny et al., 2022b). BMS-477118-induced AMPK activation could lead to a subsequent decrease in the translocation of NFκB. This effect in turn reduces the production and release of pro-inflammatory cytokines, particularly TNF-α and IL-6 (Salminen et al., 2011). It was stated that the observed decrease in TNF-α levels could potentially pay out for the noted reduction in apoptosis (Álvarez et al., 2011).

BMS-477118 was found to alleviate airway inflammation in a mouse model of acute asthma. This alleviation was achieved through the modulation of key inflammatory pathways, specifically NFκB and TLR4 (Helal et al., 2019). In addition, BMS-477118 exerted anti-inflammatory effects against nephrotoxicity induced by doxorubicin in a rat model via repressing NFκB activation and successive release of inflammatory cytokines (Mostafa et al., 2021b). Furthermore, BMS-477118 exhibited a nephroprotective effect against gentamicin-induced nephrotoxicity. This protective role is underscored by its antioxidant, anti-inflammatory, and anti-apoptotic potential (Helal et al., 2018). Our results consistently showed that an increase in AMPK activity in inflamed rat colons, in response to BMS-477118, led to further inactivation of NFκB and its target proinflammatory cytokines.

SIRT1, a NAD^+^-dependent deacetylase, plays an important role in the cellular response to inflammation and oxidative stress by modulating the expression of genes that regulate proliferation and ATP production (Yang et al., 2022b). Additionally, FOXO3a has received more research attention than the other members of the FOXO family due to its distinct and critical modulation of cell proliferation, apoptosis, metabolism, stress tolerance, and lifespan (Nho and Hergert, 2014; Fasano et al., 2019; Zhao and Liu, 2021; Bernardo et al., 2023; Cao et al., 2023). Moreover, it has been accredited that a decrease in AMPK activity is associated with a reduction in the activity of the SIRT1/FOXO3a pathway (Liu et al., 2021). Further, it was reported that AMPK plays a role in the regulation of FOXO3a activity via the activation of SIRT1. Consistent with our results, a decrease in AMPK activity in inflamed rat colons led to a further inactivation in the SIRT1/FOXO3a pathway (Thomson, 2018; An et al., 2020).

Recent research has shown that DPP4 inhibitors activate AMPK, which plays a crucial role in modulating the activity of SIRT1 and FOXO3a. The inhibition of DPP4 has been linked to improved myocardial energy metabolism and reduced endothelial senescence through the activation of the AMPK/SIRT1 signaling pathway (Furukawa et al., 2021). Additionally, DPP4 inhibitors have been found to enhance the growth and movement of rat brain microvascular endothelial cells in conditions of low oxygen and high glucose. An effect that was potentially mediated by the SIRT1 activation (Mi et al., 2019). Moreover, the activation of AMPK by DPP4 inhibitors played a role in protecting against diabetic endothelial dysfunction via the modulation of AMPK/SIRT1 signaling and the inhibition of TNF-α (Gao et al., 2020). Furthermore, linagliptin, a DPP4 inhibitor, has been shown to activate AMPK and FOXO3a, which are involved in cellular processes related to metabolic regulation and oxidative stress response (Abdelhady et al., 2024).

Consistent with our findings, it has been found that daily administration of BMS-477118 could enhance AMPK activity (Yang et al., 2018b; Chen et al., 2020). Additionally, it was previously reported that BMS-477118 regulated macrophage polarization and intensified AMPK activity in NAFLD patients (Yang et al., 2018a). Moreover, BMS-477118 ameliorated gastric mucosal damage by ethanol and displayed anti-inflammatory effects via AMPK activation (Arab et al., 2021).

The activation of AMPK induces autophagy through the negative regulation of the mammalian target of rapamycin (mTOR) protein kinase complex as well as the direct phosphorylation and activation of ULK1 (Wang et al., 2022). It was also reported that BMS-477118 alleviated the cognitive dysfunction of diabetic rats through the AMPK/mTOR pathway (Hu et al., 2023). This decrease in the mTOR expression, achieved by AMPK activation, could result in a cascade of events that ultimately lead to the removal of mTOR’s inhibitory effects on both of the key autophagy proteins, ULK1 and Beclin 1 (Yuan et al., 2018; Wang et al., 2022). Specifically, the removal of mTOR’s inhibitory effects on ULK1 could stimulate the initiation of autophagy, as ULK1 is a key protein implicated in the early stages of autophagosome formation (Nazio et al., 2013). Likewise, the removal of mTOR’s inhibitory effects on Beclin 1 could promote the nucleation of autophagosomes (Dossou and Basu, 2019). It was reported previously that DPP4 inhibition might reduce the increased risk of immediate death following a myocardial infarction associated with type 2 diabetes mellitus. This effect is possibly achieved by re-establishing the autophagic response through the modulation of ULK1 and Beclin 1 activity (Murase et al., 2015b). Additionally, the interplay between p62 and ULK1 is complex and multifaceted. p62 can bind to ULK1 in situations of proteotoxic stress triggered by an accumulation of misfolded proteins or a blockade of the proteasome. This interaction can result in the sequestration of ULK1. Subsequently, the phosphorylation status of other proteins involved in autophagy is influenced (Lim et al., 2015). This mechanism could account for the potential decrease in p62 levels in our study in response to the improvement observed in the ulcerated colons. In the present study, we demonstrated the potential of BMS-477118 to regulate autophagy in rats with chronic UC by modulating AMPK, ULK1, Beclin 1, and p62 proteins.

DPP4 inhibitors, including BMS-477118, have experienced extensive investigation viewing their impact on oxidative stress. Notably, studies have demonstrated that DPP4 inhibitors effectively reduced ROS levels in various contexts. Thereby, these agents protect against oxidative stress-induced damage (Alam et al., 2015; Bigagli et al., 2020; Singh et al., 2021; Wang X. et al., 2021; Ramos et al., 2022). A decrease in oxidative stress can lead to a related reduction in apoptosis, leading to a significant increase in cell survival. Reports have stated that BMS-477118 exerted a protective role against bone loss induced by glucocorticoids. This property could be attributed to the activation of FOXO3a and the repression of oxidative stress (Wang T. et al., 2021). Research has demonstrated that BMS-477118 can decrease the Bax/BCL-2 ratio and inhibit caspase-3 activity. By this role, a potential protective role against diabetic nephropathy was achieved (Xing et al., 2021). In parallel, treatment with BMS-477118 has been shown to elevate the levels of BCL-2 protein while reducing the levels of Bax protein in diabetic rats with renal ischemia-reperfusion (Tang et al., 2023). Additionally, BMS-477118 has been found to reduce oxidative stress and caspase-3 activation in myocardial ischemia-reperfusion injury in diabetic rats (Bisht et al., 2018). Furthermore, treatment with BMS-477118 has been displayed to significantly attenuate nephrotoxicity induced by doxorubicin. This was succeeded through the alleviation of Bax and other inflammatory mediators highlighting the potential therapeutic benefits of BMS-477118 in the management of nephrotoxicity (Mostafa et al., 2021b). However, FOXO3a has been also reported to promote apoptotic genes (Burgering and Medema, 2003). Therefore, further analysis is necessary to determine if FOXO3a is directly responsible for the reduced apoptosis observed in the inflamed colons of BMS-477118-treated chronic UC rats in the present study.

**FIGURE 8:**
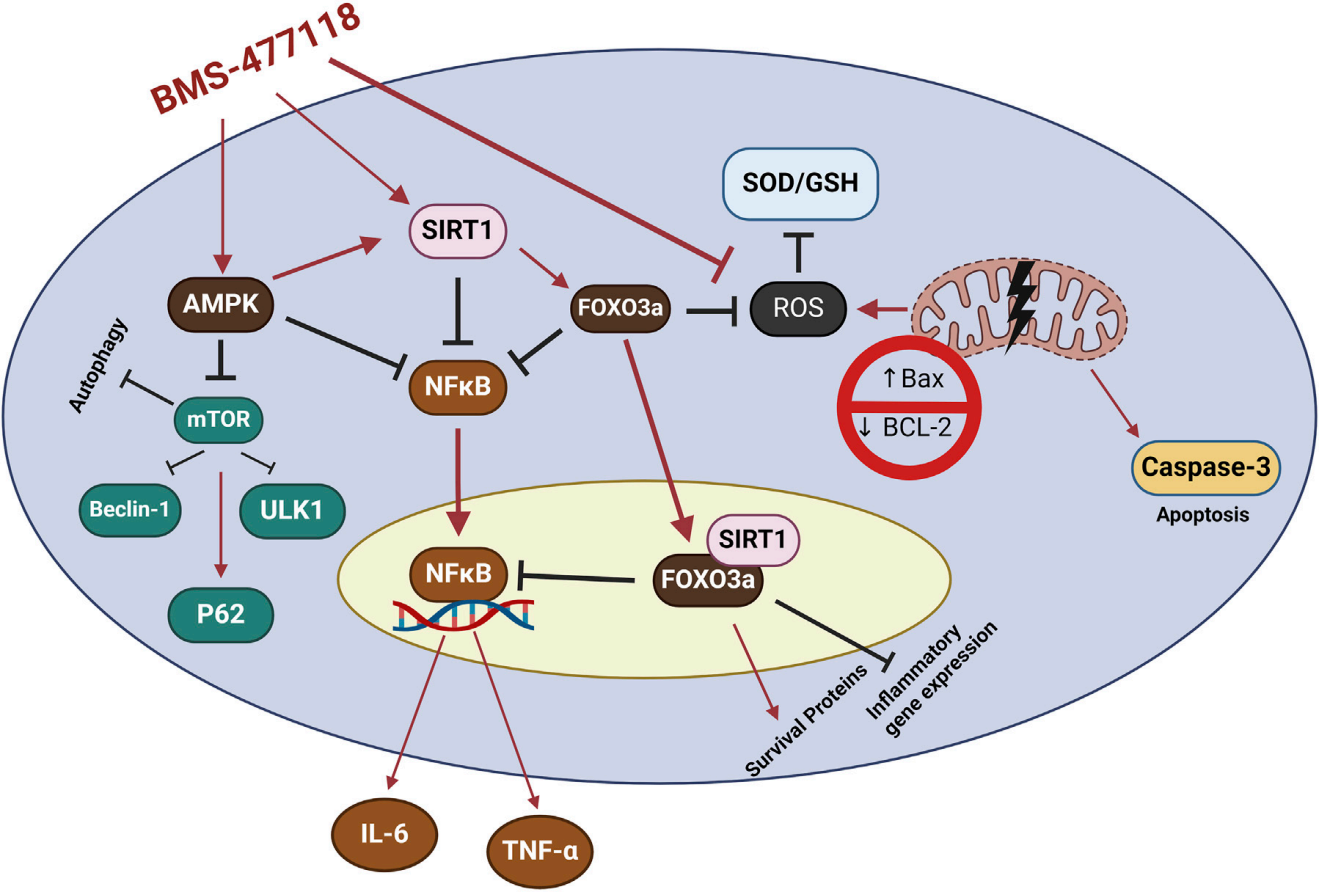
The study findings propose that the mechanism of action for BMS-477118 involves the activation of the AMPK/SIRT1/FOXO3a. Once BMS-477118 activates AMPK, the SIRT1 activity is increased. Subsequently, SIRT1 activates FOXO3a, a transcription factor involved in antioxidant defense. Through this cascade, BMS-477118 may exert its therapeutic effects in the context of colitis.

AMPK, SIRT1, and FOXO3a are part of an interconnected positive feedback loop. Active AMPK phosphorylates and activates SIRT1 by increasing cellular NAD^+^ levels, leading to the deacetylation and modulation of downstream SIRT1 targets, including FOXO3a (Cantó et al., 2009). SIRT1 directly deacetylates and activates FOXO3a (Brunet et al., 2004; Ferguson et al., 2015). In response to oxidative stress, SIRT1 and FOXO3a form a complex within cells, where SIRT1 deacetylates FOXO3a, increasing FOXO3a′s DNA binding activity. Herein, SIRT1 exerts a dual effect on FOXO3a: it enhances FOXO3a′s ability to induce resistance to oxidative stress while inhibiting its ability to induce cell death (Brunet et al., 2004). Active FOXO3a further positively regulates SIRT1 by promoting SIRT1 transcription (Yang et al., 2022a). Therefore, under stress conditions such as UC, the crosstalk between AMPK, SIRT1, and FOXO3a is critical for mounting stress resistance and facilitating cellular remodeling and repair programs. This potentially explains the observed coloprotective role of BMS-477118 in the present study (Figure 8). Our findings indicate that the activation of the interconnected positive feedback loop involving AMPK, SIRT1, and FOXO3a by BMS-477118 is associated with an improved histological appearance in injured rat colons. BMS-477118 also reduced fibrotic changes related to the chronic nature of the animal model. It alleviated macroscopic damage and disease severity, as well as the colon weight-to-length ratio. Additionally, it prevented DSS-induced weight loss and upregulated tight junction proteins. These effects, along with the reduction of oxidative stress and its anti-inflammatory, antiapoptotic, and autophagy-inducing properties, contributed to the prolonged survival of rats with chronic UC.

## 5 Conclusion

BMS-477118 provided protection against colon damage caused by the long-term use of DSS. This protection is likely due to BMS-477118s ability to activate the AMPK/SIRT1/FOXO3a signaling pathway in inflamed colons of DSS-challenged rats. These findings suggest that the AMPK/SIRT1/FOXO3a signaling pathway could be a new target for treating UC, decreasing inflammation and intestinal fibrosis. Further research into the therapeutic potential of this pathway is needed. Additionally, ongoing investigations into the therapeutic potential of BMS-477118 and other DPP4 inhibitors show promise for developing new treatment approaches for various medical needs.

## Data Availability

The original contributions presented in the study are included in the article/supplementary material, further inquiries can be directed to the corresponding authors.
